# A Case Report of Acute Pancreatitis in Food-Induced Anaphylaxis

**DOI:** 10.7759/cureus.71017

**Published:** 2024-10-07

**Authors:** Jennifer Wiese, Bassel Dakkak, Onyinye Ugonabo, Mohammed El-Dallal, Wesam Frandah

**Affiliations:** 1 Internal Medicine, Marshall University Joan C. Edwards School of Medicine, Huntington, USA; 2 Internal Medicine, School of Medicine, Marshall University Hospital, Huntington, USA; 3 Gastroenterology, Marshall University Joan C. Edwards School of Medicine, Huntington, USA; 4 Internal Medicine/Gastroenterology, Marshall University Joan C. Edwards School of Medicine, Huntington, USA

**Keywords:** acute pancreatitis, allergy and anaphylaxis, food-induced anaphylaxis, glp-1 receptor agonists, steroid-induced pancreatitis

## Abstract

Food allergy-induced pancreatitis is a rare condition that presents unique diagnostic challenges. While acute pancreatitis (AP) is typically linked to factors such as gallstones, alcohol consumption, metabolic issues, medications, and autoimmune conditions, food allergies are seldom considered a potential cause. Diagnosing food allergy-induced pancreatitis often requires a high index of suspicion and the exclusion of more common causes of pancreatitis.

Here we report a 54-year-old female patient who presented at the emergency department (ED) experiencing an anaphylactic reaction to food. After receiving treatment for anaphylaxis, she developed acute abdominal pain 12 hours later. A CT scan of the abdomen indicated AP. The patient was managed with supportive care, including analgesics and intravenous fluids, and did not experience any further complications. Other potential causes and risk factors for AP were ruled out or deemed unlikely. This case highlights the significance of diagnosing AP, particularly food allergy-induced pancreatitis in patients with anaphylaxis. Early detection and early initiation of therapy can subsequently reduce morbidity and mortality.

## Introduction

Food allergies are increasingly recognized as a major concern, affecting about 10.8% of adults in the US [1]. About 203,000 ED visits each year have been linked to food-related acute allergic reactions [2]. It is well known how they can be potentially life-threatening and may lead to anaphylaxis and death, but they are rarely reported to be the cause of other health problems. The exaggerated reaction between the body and the allergen can impact other body systems and lead to significant health issues beyond typical allergic symptoms [3]. While pancreatitis is commonly associated with toxic, metabolic, or mechanical causes, food allergies are not listed among its etiologies [4]. However, there are rare, documented cases suggesting a connection [3,5-10]. This case report further suggests the presence of a causative relationship between food allergies and pancreatitis. Our case will enrich the literature on this matter and will highlight how food allergy-induced pancreatitis should be considered in idiopathic pancreatitis cases, which could lead to more targeted diagnoses and improved management strategies.

## Case presentation

A 54-year-old female with a history of asthma and hypothyroidism was brought into the ED by emergency medical service following an anaphylactic reaction to food. She reported feeling nauseous after eating steak alfredo followed by vomiting and the onset of severe pruritus, hives, shortness of breath, chest tightness, and loose bowel movement along with a pre-syncopal episode. The patient disclosed known allergies to oxycodone-acetaminophen, latex, milk, azithromycin, and contrast. She denied any previous episode of anaphylaxis and has not had any recent change of medication. Home medications include albuterol, ergocalciferol, fluoxetine, fluticasone, phentermine, sumatriptan, levothyroxine, and semaglutide, a glucagon-like-peptide-1 receptor agonist (GLP-1 RA) for weight loss. The patient drinks alcohol occasionally but denies cigarette smoking or illicit drug use. In the ED, the patient was hypotensive with a blood pressure of 75/66, and a heart rate of 75 beats per minute. She received 1 liter (L) of lactated Ringer's (LR), intramuscular epinephrine, diphenhydramine, and methylprednisolone. Initial laboratory findings were significant for white blood count of 11 K/µL (reference 4.5-10 K/µL), hemoglobin 16.3 g/dl (12-15 g/dl), platelet 464 K/µL (150-450 K/µL), elevated lymphocytes 4.9% (1.0-4.8%), and CRP <0.1 mg/dl (0.1-0.5 mg/dL) (Table 1). 

**Table 1:** Laboratory values on admission and throughout the hospitalization. -: not available

Laboratory tests	Lab results on admission	Lab results 12 hours after admission	Day 2	Day 3	Day 4	Day 5	Reference range
WBC	11	8.8	23.5	20	14.6	10.5	4.5-10 K/µL
Hgb	16.3	14.4	13	12.7	11.8	12.6	12.0 - 15.0 g/dL
Creatinine	1.1	1	0.8	0.7	0.6	0.6	0.7 - 1.3 mg/dL
Protein total	6.9	-	6.7	-	6.8	7	6.0 - 8.3 g/dL
Albumin	3.8	-	4	3.6	-	3.5	3.5 - 5.7 g/dL
Bilirubin total	0.4	-	0.7	0.9	-	0.5	0.3 - 1.0 mg/dL
Bilirubin direct	-	-	0.1	0.3	-	-	0.0 - 0.2 mg/dL
Alkaline phosphatase	64	-	61	65	-	82	34 - 104 U/L
SGPT (ALT)	15	-	12	9	-	10	7 - 52 U/L
SGOT (AST)	21	-	15	12	-	13	13 - 39 U/L
Amylase	-	246	159	-	-	30	30-110 U/L
Lipase	-	558	265	-	-	33	11 - 82 U/L

**Figure 1:**
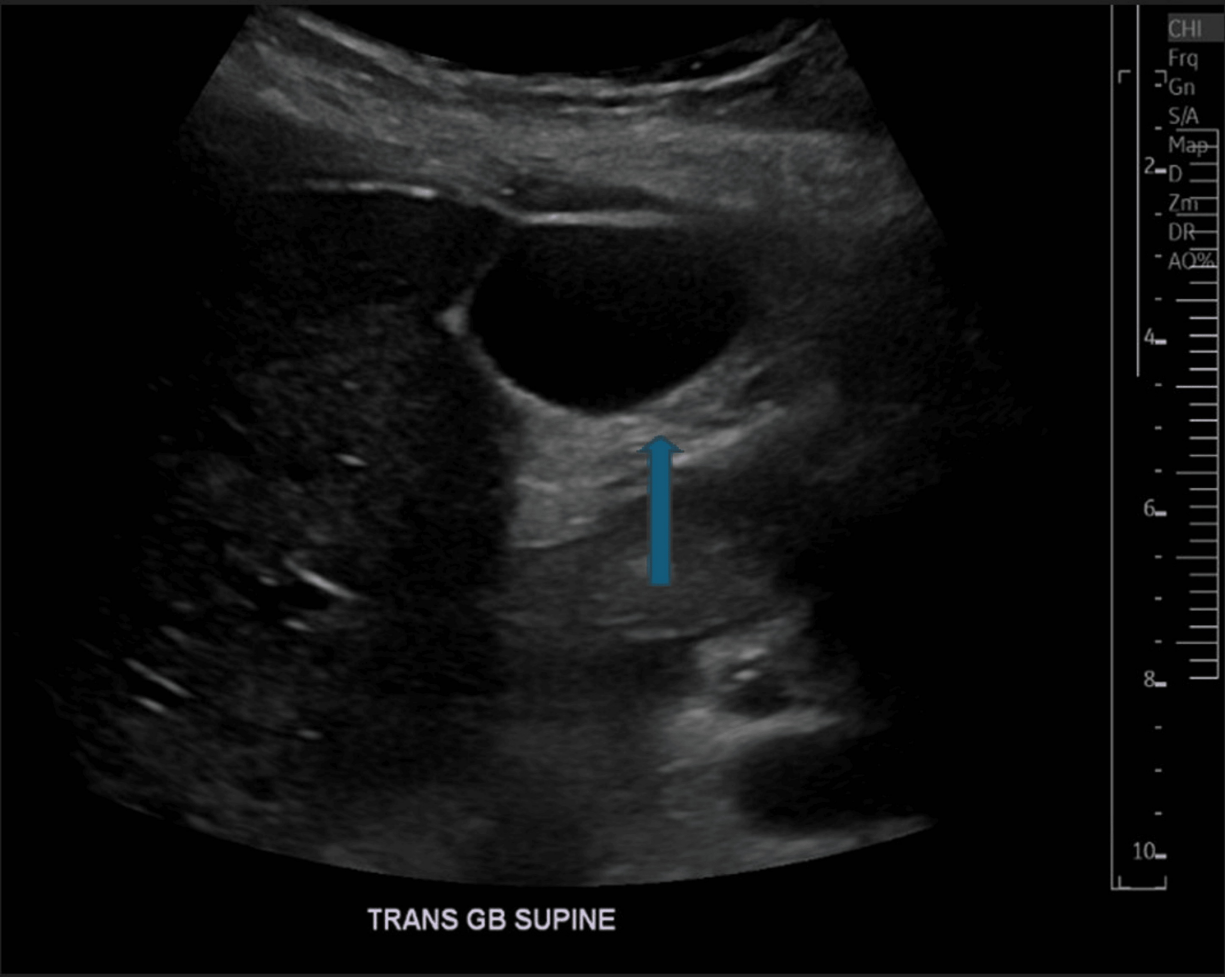
Ultrasound abdomen showing normal gallbladder with no gallstones (blue arrow).

**Figure 2:**
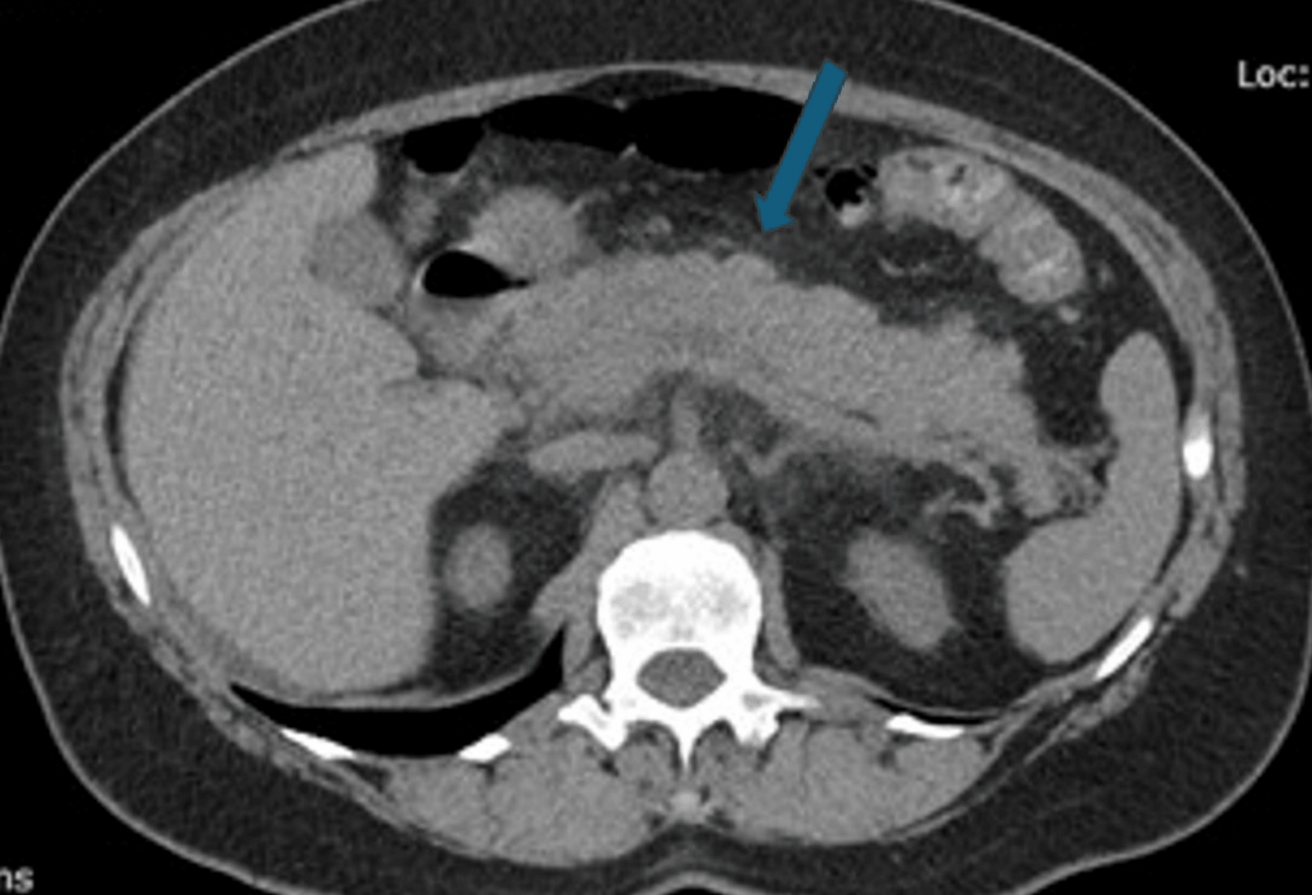
CT abdomen displaying diffuse fat stranding and edema around the pancreatic body, head, and uncinate process (blue arrow).

Her vitals improved with BP of 102/75 mmHg, HR of 87 bpm, and respiratory rate of 14 per minute. The patient was continued with another liter of LR and was admitted for further monitoring of her symptoms (severe pruritus, hives, shortness of breath, chest tightness, hypotension). About 12 hours later, the patient started experiencing abdominal pain. Immediate laboratory tests and imaging studies were conducted. Ultrasound of the abdomen showed no gallstones (Figure 1). Computed tomography of the abdomen displayed diffuse fat stranding and edema across the pancreatic body, head, and uncinate process, concerning for acute pancreatitis (AP) (Figure 2). There was no definite focus on hyperattenuating infectious necrosis, no appreciable cyst or pseudocyst. Edematous changes were described in the adjacent duodenum and colon at the hepatic flexure. Repeat labs showed significantly elevated lipase of 558 units/L (11-82 units/L). Other values are illustrated in Table 1. 

The patient was managed supportively for AP, including analgesics and continuous intravenous fluid with LR at 1.5 ml/kg/h for 24 hours. She experienced no further complications. Her abdominal pain improved, and she began to tolerate diet. Based on her presentation, imaging results, and response to treatment, other causes of AP were considered unlikely, leading to a suspicion of allergy-induced pancreatitis. No additional interventions or diagnostics were necessary. After five days, she was deemed stable for discharge with scheduled follow-up appointments.

## Discussion

This article reports a case of anaphylaxis-induced AP. The patient’s initial clinical presentation of anaphylaxis improved significantly with supportive therapy. Twelve hours later, her condition was complicated with AP. The diagnosis of AP was confirmed by clinical findings, laboratory, and CT-abdomen results.

The incidence of AP is increasing worldwide. This is either due to increased metabolic syndrome associated with an increased risk of gallstones and hypertriglyceridemia or an increase in early detection [11,12]. Often the etiology of AP is multifactorial. Though the most common etiological factors are biliary stone, ethanol, idiopathic, and triglyceride, the less frequent causes are drug-induced, autoimmune, infectious, neoplastic, traumatic, and vascular. AP has rarely been linked to food allergy and anaphylaxis [3,5-10].

AP is categorized according to the Atlanta classification into interstitial edematous pancreatitis and necrotizing pancreatitis [13,14]. The severity of the disease is classified into three types: mild, moderate, and severe. In mild AP, there are no local or systemic complications, nor is there any organ failure (such as kidney or respiratory failure, infection, pseudocyst formation, or diabetes). Moderately severe AP is characterized by local complications with or without organ failure lasting less than 48 hours. Severe AP involves persistent organ failure for more than 48 hours, affecting one or more organs. Furthermore, various scoring systems have been developed to assess the severity of the condition, with the most commonly used tools being the Bedside Index of Severity in Acute Pancreatitis (BISAP) and the Acute Physiology and Chronic Health Evaluation (APACHE) II. These tools have proven effective in predicting the severity of the illness according to the revised Atlanta classification [15].

To this date, the underlying pathomechanism of food anaphylaxis-induced AP is poorly understood. However, several pro-inflammatory cells such as neutrophils, eosinophils, mast cells, dendritic cells, monocytes, macrophages, T cell subsets, cytokines, and chemokines are known to have critical roles in promoting food-mediated allergic responses in pancreatitis [16,17]. In one case report of recurrent pancreatitis secondary to food allergy, inflammatory changes to the ampulla of Vater in an endoscopic exam had been documented [9]. On immunohistochemical staining against human mast cell tryptase, many mast cells in the mucosa and submucosa were found. The authors postulated inflammatory changes obstructing the ampulla of Vater, leading to reflux of bile into the pancreatic duct and improper activation of zymogen could be the cause of food anaphylaxis-induced pancreatitis [9]. Anaphylaxis can also cause vasodilation and increased vascular permeability, leading to hypotension and reduced blood flow to the pancreas. This can compromise pancreatic perfusion and contribute to tissue injury.

Drug-induced AP was also considered as differential in this case. One of the possible offending agents for AP was steroids. Our patient received methylprednisolone for anaphylaxis upon her presentation. Based on the existing literature, steroid-induced pancreatitis develops within 4-14 days after initial exposure [18]. Since our patient received steroids 12 hours before her initial symptoms of AP, steroid-induced pancreatitis was unlikely the cause of her AP.

Another possible contributing factor for AP addressed in our case was the GLP-1 agonist, which our patient has been taking for years. GLP-1 is a hormone released from the intestinal enteroendocrine L cells of the ileum and colon that leads to glucose-dependent insulin secretion from the pancreas. GLP-1 stimulates receptors that are expressed in pancreatic islet and exocrine duct cells. It has been suggested that stimulation of these receptors by GLP-1 agonists may lead to hyperplasia of the cells, potentially obstruct the smaller ducts, and subsequently lead to chronic low-grade or acute inflammation [19,20].

According to the study by Gorgojo-Martinez and colleagues, nausea, vomiting, diarrhea, and constipation are the most frequently associated adverse effects of GLP-1 RA among gastrointestinal side effects [21]. AP associated with this GLP-1 RA was generally low (<1%) [22]. However, in two systemic reviews and meta-analyses that included more than 9000 and 300,000 patients with diabetes treated with GLP-1 RA, they found no association between GLP-1 agonist therapy and AP [22,23]. Given the patient’s acuity of symptoms and low risk of AP associated with GLP-1 RA, medication-induced AP is unlikely in this scenario. Due to guidelines recommending discontinuation of GLP-1 agonist use, if AP develops, the medication was stopped in our patient [24].

## Conclusions

In conclusion, this case underscores the importance of promptly recognizing and treating anaphylaxis, particularly in patients with pre-existing conditions such as allergic asthma. The patient's presentation and rapid deterioration emphasize the necessity of thorough history-taking and vigilance in emergency situations. Effective management, including timely administration of epinephrine and supportive care, is crucial in alleviating the potentially life-threatening consequences of anaphylactic reactions. This case reinforces the need for healthcare providers to remain aware of food allergies and their implications for patient safety.

Furthermore, this case emphasizes the significance of diagnosing AP in patients experiencing anaphylaxis. Timely diagnosis, assessment of severity, and appropriate management can help reduce morbidity and mortality. The outcomes of AP are affected by risk stratification, fluid, and nutritional management, as well as follow-up care and risk reduction strategies. Recognizing food allergies as a risk factor and potential cause of AP may facilitate the removal of the offending agent and possible desensitization to the allergen.
